# CD49d is a disease progression biomarker and a potential target for immunotherapy in Duchenne muscular dystrophy

**DOI:** 10.1186/s13395-015-0066-2

**Published:** 2015-12-10

**Authors:** Fernanda Pinto-Mariz, Luciana Rodrigues Carvalho, Alexandra Prufer De Queiroz Campos Araujo, Wallace De Mello, Márcia Gonçalves Ribeiro, Maria Do Carmo Soares Alves Cunha, Pedro Hernan Cabello, Ingo Riederer, Elisa Negroni, Isabelle Desguerre, Mariana Veras, Erica Yada, Yves Allenbach, Olivier Benveniste, Thomas Voit, Vincent Mouly, Suse Dayse Silva-Barbosa, Gillian Butler-Browne, Wilson Savino

**Affiliations:** Laboratory on Thymus Research, Oswaldo Cruz Foundation, Rio de Janeiro, Brazil; Institute of Pediatrics, Federal University of Rio de Janeiro, Rio de Janeiro, Brazil; Sorbonne Universités, UPMC Univ Paris 06, UM76, INSERM U974, CNRS FRE3617, Center for Research in Myology, 47 boulevard de l’Hopital, Paris, 75651 France; Laboratory on Human Genetics, Oswaldo Cruz Institute, Oswaldo Cruz Foundation, Rio de Janeiro, Brazil; INSERM U-E10, Necker Hospital, Paris, France; Service de Médecine Interne 1, Université Pierre et Marie Curie, Paris, France; Department of Clinical Research, National Cancer Institute (INCA), Rio de Janeiro, Brazil

**Keywords:** Duchenne muscular dystrophy, Inflammation, CD49d, T lymphocytes, Predictive biomarker, Immunotherapy

## Abstract

**Background:**

Duchenne muscular dystrophy (DMD) is caused by mutations in the dystrophin gene. The immune inflammatory response also contributes to disease progression in DMD patients. In a previous study, we demonstrated higher levels of circulating CD49dhi and CD49ehi T cells in DMD patients compared to healthy control. DMD patients are clinically heterogeneous and the functional defect cannot be correlated with genotype. Therefore, it is important to be able to define reliable noninvasive biomarkers to better define the disease progression at the beginning of clinical trials.

**Results:**

We studied 75 DMD patients at different stages of their disease and observed that increased percentages of circulating CD4^+^CD49d^hi^ and CD8^+^CD49d^hi^ T lymphocytes were correlated with both severity and a more rapid progression of the disease. Moreover, T^+^CD49d^+^ cells were also found in muscular inflammatory infiltrates. Functionally, T cells from severely affected patients exhibited higher transendothelial and fibronectin-driven migratory responses and increased adhesion to myotubes, when compared to control individuals. These responses could be blocked with an anti-CD49d monoclonal antibody.

**Conclusion:**

CD49d can be used as a novel biomarker to stratify DMD patients by predicting disease progression for clinical trials. Moreover, anti-CD49d peptides or antibodies can be used as a therapeutic approach to decrease inflammation-mediated tissue damage in DMD.

**Electronic supplementary material:**

The online version of this article (doi:10.1186/s13395-015-0066-2) contains supplementary material, which is available to authorized users.

## Background

Duchenne muscular dystrophy (DMD) is the most common genetic muscular dystrophy, affecting 1 in 5000 male births. It is caused by mutations in the dystrophin gene, leading to functional loss or absence of the protein [1, 2]. The results of a recent phase 3 clinical trial using exon-skipping strategies failed to show significant functional improvement [3, 4] probably due to the clinical heterogeneity of the patients that is a hallmark of the disease [5]. This emphasizes the urgent need to define reliable noninvasive biomarkers to better define these patient populations at the beginning of the trial and monitor the results of corrective strategies.

It is known that in DMD, there is an inflammatory process following muscular necrosis, which leads to fibrotic remodeling [6]. In this context, it has been demonstrated that there is a correlation between the absence of B and T lymphocytes and a decrease in fibrosis in the SCID/mdx mouse [7]. Fibrogenetic growth factors and their receptors are upregulated and localized with inflammatory cells in muscles of DMD patients [8]. More recently, it has been demonstrated that osteopontin, the ligand for the integrin VLA-4, is upregulated in the muscle fibers and inflammatory infiltrates in DMD patients, suggesting that it is involved in both fibrosis and inflammation [9]. A dysregulation in extracellular matrix (ECM) expression has also been demonstrated in DMD patients [10–12], together with increased expression of ECM receptors on inflammatory cells near regions of necrosis in the mdx model [13]. These data suggest that ECM-mediated cell interactions contribute to the migration of cells to the site of muscle damage, triggering and maintaining local inflammation and fibrosis.

We previously found that DMD patients exhibited higher relative numbers of CD4^+^ and CD8^+^ T cells expressing higher levels of the alpha-4 chain of the integrin VLA-4 (CD49d^hi^) and alpha-5 chain of the integrin VLA-5 (CD49e^hi^), both of which are fibronectin receptors [14]. In the present study, we investigated if those subpopulations are correlated with disease severity and disease progression in DMD patients. We demonstrate that CD49d can be used as a biomarker to monitor both severity and progression of disease in DMD patients. In addition, ex vivo cell migration experiments strongly suggest that CD49d participates to the migration of inflammatory cells into the muscle.

To our knowledge, this is the first study that proposes a reliable circulating biomarker that can be used to stratify DMD patients into homogeneous groups thereby improving the power of significance of results of clinical trials. Finally, these results suggest that in addition CD49d could be used as a therapeutic target to slow down disease progression in DMD boys.

## Methods

### Patients

We enrolled 75 patients from the Pediatrics Institute, Federal University of Rio de Janeiro (IPPMG/UFRJ), Brazil (Additional file 1: Table S1), for whom the genetic and/or histological diagnosis of DMD had been confirmed. They were divided into three groups according to their ability to walk: 10 m in less than 10 s (speed > 1 m/s; *n* = 19), 10 m in 10 s or more (speed ≤ 1 m/s; *n* = 25), and unable to walk (*n* = 31). These groups were further subdivided according to the age at which they had stopped walking: before or after 10 years of age (rapid progression, *n* = 16, and slow progression, *n* = 15, respectively). A control group (*n* = 14) comprised age-/sex-matched healthy volunteers.

We also developed a prospective study of CD49d levels on T cells in DMD patients who were able to walk at a speed ≤1 m/s and were followed until they lost their ability to walk. If such a loss occurred before 10 years of age, this was considered rapid progression, whereas after 10 years of age, it was considered slow progression.

We also evaluated biopsies from DMD patients who had lost ambulation before (*n* = 4) or after (*n* = 5) 10 years of age. The biopsies were obtained at the beginning of the disease as part of the diagnostic procedure, at Necker Children’s Hospital, Paris, France (Additional file 2: Table S2). As controls, we used three age-/gender-matched muscle samples from children undergoing orthopedic surgery for non-muscle-related reasons.

Twenty-two IBM patients were enrolled for a natural history study of IBM, and their diagnosis and clinical characteristics are described [15]. Peripheral blood mononuclear cells (PBMCs) of IBM patients were compared to healthy subjects (*n* = 22), who were matched for sex and age, free of inflammatory/autoimmune diseases, past history of cancer or active cancer, and were not receiving any immunosuppressive or immunomodulatory drug. The institutional review board approved the study protocol, also for ancillary studies on their blood samples [16].

Procedures have been approved by the ethical committees of the IPPMG/UFRJ (Research and Ethical Committee of IPPMG/UFRJ—*Comitê de Ética e Pesquisa do Instituto de Puericultura e Pediatria Martagão Gesteira/Universidade Federal do Rio de Janeiro*; reference number 06/06), CPP Ile de France (reference number 14.323), and the *Ministère de la Recherche* and Cochin Hospital Cell Bank, Paris, agreement number DC-2009-944. All the patients and/or parents gave written individual informed consent to participate in the study.

### In situ immunofluorescence and cytofluorometry

General features of antibodies are presented in Additional file 3, Table S3. Sequential gating of cells for CD49d evaluation is illustrated in Additional file 4, Figure S1. For cytofluorometry, we used fluorochrome-labeled monoclonal antibodies with specificities for CD3, CD4, CD8, CD11a, CD14, CD19, CD49a, CD49d, CD49e, or CD49f. Isotype-/fluorochrome-matched unrelated antibodies were also Pharmingen reagents. Flow cytometry assays were carried out as described previously [14]. To characterize the inflammatory infiltrate in the muscle biopsies, we used anti-CD3, anti-CD4, anti-CD8, anti-CD49d, and anti-HLA-DR monoclonal antibodies (mAb). Nuclei were visualized using DAPI (Sigma-Aldrich, St. Louis, USA). Secondary goat anti-mouse or goat anti-rabbit antibodies conjugated to fluorochromes (Alexa Fluor 594, Alexa Fluor 488, Streptavidin Cy5—in the case of biotinylated secondary antibody) were used to reveal primary antibodies.

In situ immunofluorescence analyses were made on frozen sections of muscle biopsies as described [17]. We performed immunostaining for simultaneous detection of CD3, CD4, or CD8; HLA-DR; and CD49d. In each muscle biopsy, all the inflammatory fields were recorded. We have also performed experiments with isotype-matched unrelated reagents in the same muscle samples obtained from DMD and healthy control subjects: no background staining was observed. We performed immunostainings in samples from patients with IBM and polymyositis, and the staining patterns for CD3, CD4, and CD8 matched those described in the literature.

Images were analyzed with Metamorph software (Molecular Devices, Toronto, Canada). The absolute numbers of CD8^+^ and CD4^+^ cells in situ were determined in all inflammatory fields, defined by the presence of cell clusters identified with DAPI nuclear staining.

### Cell migration assays

The migratory responses of T lymphocytes through fibronectin or endothelial cells were measured using 5-μm pore size *Transwell* chambers (Costar; Corning). For fibronectin-driven migration, the insert membranes were coated and blocked as described [17]. For transendothelial cell migration, 10^5^ human umbilical vein endothelial cells (Promo Cell, Heidelberg, Germany) were added onto the insert membranes of transwell plates. After 24 h, the cultures were confluent and the inserts were washed with RPMI-1640. In both assays, 10^6^ PBMCs were placed in the upper chamber and left to migrate for 16 h. Migrating cells were phenotyped by flow cytometry. For the blocking assay, 10^6^ cells were pretreated for 10 min with 10 μl of purified anti-CD49d mAb and migrated as described above. In the case of blockage of adhesion by specific antibodies, data are presented as a percentage of the migration values obtained following pretreatment with unrelated antibodies, applied on cells from the same subject. In cell migration experiments, data was normalized to measure the percentage of input, using the following formula:

$$ \begin{array}{l}\%\;\mathrm{input}=\left(\mathrm{number}\;\mathrm{o}\mathrm{f}\;\mathrm{migrating}\;\mathrm{cells}\;\mathrm{o}\mathrm{f}\;\mathrm{a}\;\mathrm{given}\;\mathrm{phenotype}\times 100\right)/\mathrm{total}\;\mathrm{number}\;\mathrm{o}\mathrm{f}\;\mathrm{cells}\;\mathrm{o}\mathrm{f}\;\mathrm{a}\;\mathrm{given}\;\mathrm{phenotype}\;\\ {}\mathrm{a}\mathrm{llowed}\;\mathrm{t}\mathrm{o}\;\mathrm{migrate}\end{array} $$

### Lymphocyte adhesion to myotubes

Human myoblasts were cultured in Ham’s F-10 medium [18]. Differentiation was induced as described [19]. One million PBMCs from healthy individuals or DMD patients were allowed to adhere onto myotubes for 2 h at 37 °C and washed to remove non-adherent PBMCs. Adherent cells were counted and phenotyped. Unrelated IgG1 or anti-CD49d mAb (1 μg/ml) was added in PBMC suspensions before co-culturing with myotubes. In the case of adhesion blockage by specific antibodies, data are presented as percentages of the values obtained with unrelated antibodies, applied on cells from the same subject.

### Statistics

For continuous data, groups were compared by the Mann-Whitney test (for two groups) or Kruskal-Wallis test followed by Dunn’s multiple comparison test (for more than two groups). Statistical analyses were performed using GraphPad Prism software version 5 for Windows. Differences were considered statistically significant when *p* values were ≤0.05.

## Results

### High numbers of CD49d^hi^ T cells are correlated with disease progression

We first evaluated the membrane expression levels of various integrin α subunits on circulating T cells, comparing a large cohort of DMD patients with healthy individuals (Table 1). We found higher relative numbers of CD49d^hi^CD4^+^ (*p* = 0.007) and CD8^+^ T lymphocytes (*p* = 0.009) in DMD patients, although they exhibited similar numbers of CD4^+^ or CD8^+^ T cells/mm in the blood compared to controls. This was specific to T cell subsets, since CD49d expression levels were similar in CD19^+^ B lymphocytes and CD14^+^ monocytes, when comparing healthy and DMD subjects (Additional file 5: Figure S2).

**Table 1:** Higher relative numbers of circulating CD4^+^ and CD8^+^ T cell subsets expressing high densities of CD49d in patients with Duchenne muscular dystrophy

T cell subpopulation	Relative cell number (mean ± SD)^a^		*p* value
	Healthy	DMD	
CD4^+^CD49a^hi^	3.66 ± 2.80	4.34 ± 3.18	0.75
CD8^+^CD49a^hi^	3.81 ± 3.25	4.02 ± 3.66	0.93
CD4^+^CD49d^hi^	23.25 ± 5.86	*29.72 ± 8.66*	*0.007*
CD8^+^CD49d^hi^	26.28 ± 5.89	*34.66 ± 12.00*	*0.009*
CD4^+^CD49e^hi^	34.95 ± 9.28	34.97 ± 7.70	0.87
CD8^+^CD49e^hi^	31.10 ± 8.17	34.04 ± 13.42	0.46
CD4^+^CD49f^hi^	30.53 ± 7.60	27.85 ± 9.37	0.36
CD8^+^CD49f^hi^	18.73 ± 5.37	18.34 ± 7.75	0.65
CD4^+^CD11a^hi^	22.71 ± 14.23	19.30 ± 12.80	0.60
CD8^+^CD11a^hi^	38.31 ± 11.39	44.64 ± 11.30	0.33

^**a**^Data are presented as relative cell numbers of T cell subsets expressing high levels of a given integrin subunit. Numbers in italics illustrate statistically significant differences between normal subjects and Duchenne muscular dystrophy (DMD) patients, with corresponding *p* values

**Fig. 1:**
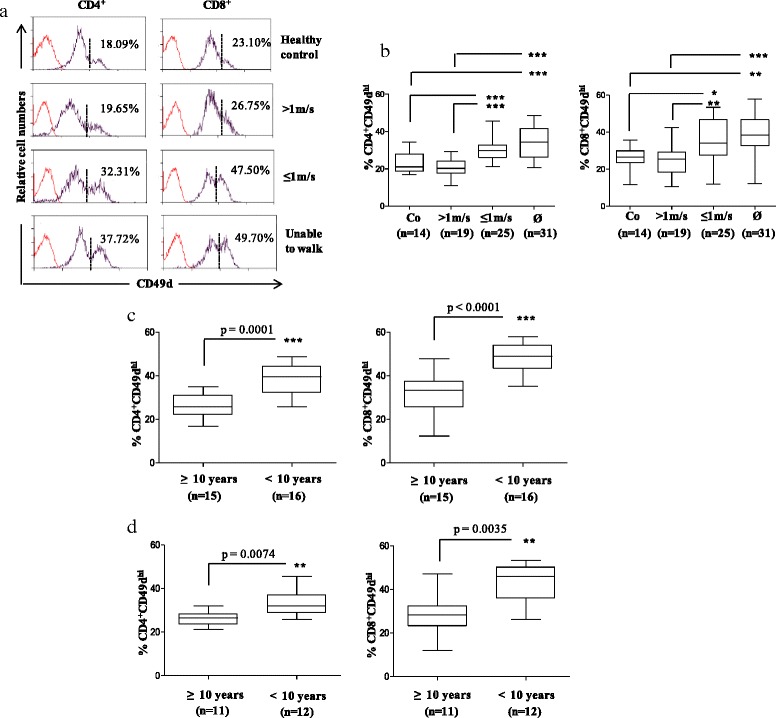
Higher relative numbers of CD49d^hi^ T cells correlate with rapid disease progression. **a** CD49d membrane expression in CD4^+^ and CD8^+^ T cell subsets. The values correspond to the percentages of the T cell subpopulation with a high CD49d expression (CD49d^hi^ T). *Dashed bars* define high versus low CD49d expression, and the *red curves* represent isotype-matched antibody. **b** Relative numbers of CD49d^hi^CD4^+^ and CD49d^hi^CD8^+^ T cells in different groups of DMD patients organized according to their disease progression, as well as healthy controls. **c** Retrospective study of wheelchair-bound patients organized according to the age when they lost ability to walk. **d** Prospective study in patients able to walk 1 m/s or less, organized according to the age when they became wheelchair bound. The number of DMD patients analyzed in each group appears in *parentheses*. **b** *p < 0.05, **p < 0.01, ***p < 0.001

When DMD patients were split into different sub-groups, able to walk relatively fast (more than 1 m/s), still able to walk but more severely affected (1 m/s or less), and those who were no longer able to walk, the relative numbers of CD49d^hi^CD4^+^ and CD49d^hi^CD8^+^ T cells were significantly higher in the more severe DMD patients, as compared to the groups ≤1 m/s and unable to walk (Fig. 1a, b). No significant difference was observed between patients that could walk faster than 1 m/s with healthy controls.

In a retrospective study of wheelchair-bound DMD patients, we observed higher percentages of CD49d^hi^CD4^+^ and CD49d^hi^CD8^+^ T cells in patients who had lost ambulation before 10 years of age (rapid disease progression), compared to those who had become wheelchair bound at or after 10 years of age (slow disease progression) (Fig. 1c). These findings clearly indicate that higher numbers of CD49d^hi^ T cells correlate with rapid disease progression.

In a prospective study of 23 DMD patients followed until loss of ambulation, we found that a significantly higher number of CD49d^hi^CD4^+^ and CD49d^hi^CD8^+^ T cells were observed at the beginning of the study in the group of patients who subsequently lost ambulation before 10 years of age (*n* = 12), as compared to those in the group who lost ambulation later (*n* = 11). These results confirm that the relative number of circulating CD49d^hi^ T cells can predict disease progression (Fig. 1d). If we look for the relative numbers of both subpopulations, we observed that DMD patients with CD49d^hi^CD4^+^ ≥ 31.91 % and CD49d^hi^CD8^+^ T cells ≥ 45.90 % have 100 % chance to lose ambulation before 10 years of age (sensitivity of 41 %; *x*^2^ = 5.85; *p* = 0.01). This will be very important when carrying out clinical trials for new drugs in the future.

Since no statistical difference in levels of circulating CD49d^hi^ T cells could be observed between ≤1 m/s ambulating versus non-ambulating patients, we thus split the patients into two groups (slow progressors: unable to walk with 10 years or more, and fast progressors: unable to walk before 10 years) and check the numbers of CD49d^hi^ T cells just as a function of disease progression. For the slow progressors, no significant difference was found between ≤1 m/s (*n* = 11) and non-ambulating (*n* = 15) patients. For the fast progressors, we observed higher numbers of CD49d^hi^ T cells in non-ambulating patients (*n* = 16) when compared to ≤1 m/s patients (*n* = 12) (Additional file 6: Figure S3).

We performed the same analyses in a longitudinal study, following the same patient over time (≤1 m/s and unable to walk). Again, we observed higher numbers of CD49d^hi^CD4^+^ T cells when the patient became unable to walk comparing with ≤1 m/s only in the group of fast progressors (*n* = 4) (Additional file 6: Figure S3).

To investigate whether CD49d^hi^ is also a common biomarker in patients with inflammatory disorders of the skeletal muscle, we investigated the relative numbers of CD49d^hi^ T cells from patients with inclusion body myositis (IBM), an example of an inflammatory disorder of the skeletal muscle [20]. It is important to note that no difference was observed when IBM patients were compared to healthy controls (Table 2), further reinforcing the role of the relative numbers of CD49d^hi^ T as a biomarker for DMD severity in patients with established genetic diagnosis.

**Table 2:** No difference was observed in the relative numbers of circulating CD4^+^ and CD8^+^ T cell subsets expressing high densities of CD49d in patients with IBM and healthy controls

T cell subpopulation	Relative cell number (mean ± SD)^a^		*p* value	Number of individuals	
	Healthy	IBM		Healthy	IBM
CD4^+^CD49d^hi^	35.24 ± 13.10	34.23 ± 18.25	0.44	21	23
CD8^+^CD49d^hi^	44.89 ± 14.66	43.92 ± 14.59	0.60	17	20

^**a**^Data are presented as relative cell numbers of T cell subsets expressing high levels of the CD49d integrin subunit. No significant differences (*p* > 0.05) were seen in both T cell subpopulations when healthy patients were compared to IBM individuals

### CD49d^+^-activated T lymphocytes in inflammatory infiltrates

**Fig. 2:**
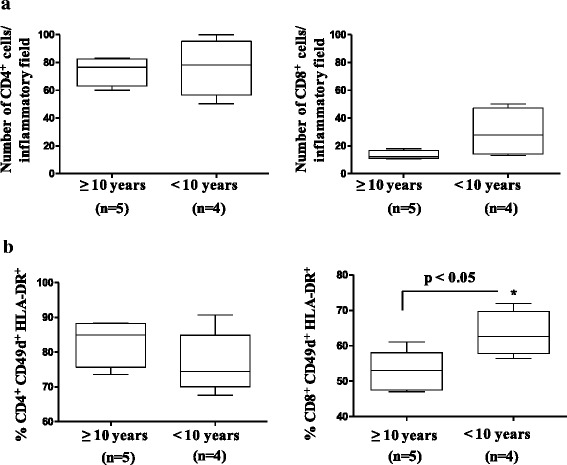
Higher numbers of activated CD8^+^CD49d^+^ T cells in muscle of DMD patients with rapid progression. **a** Number of CD4^+^ and CD8^+^ T lymphocytes per inflammatory infiltrate. **b** Relative number of T cells simultaneously expressing CD49d and the T cell activation marker HLA-DR. The biopsies were performed at the beginning of the disease, and patients were divided into two groups according to the age when they became wheelchair bound. The number of DMD patients analyzed in each group appears in *parentheses*

We investigated CD49d in T cells within the skeletal muscle of nine DMD patients. Initial analysis showed no difference in the numbers of CD4^+^ T cells and a trend to a higher number of CD8^+^ T lymphocytes within the inflammatory infiltrate (Fig. 2a) in the muscle of rapid-progressing DMD patients. However, when we applied HLA-DR immunostaining as a T cell activation marker, we detected significantly higher numbers of tissue-resident CD49d^+^HLA-DR^+^CD8^+^ T lymphocytes (but not CD49d^+^HLA-DR^+^CD4^+^ T cells) in patients who had a rapid disease progression (Fig. 2b) (*p* < 0.05).

### CD49d-mediated T cell migratory responses correlate with disease progression

**Fig. 3:**
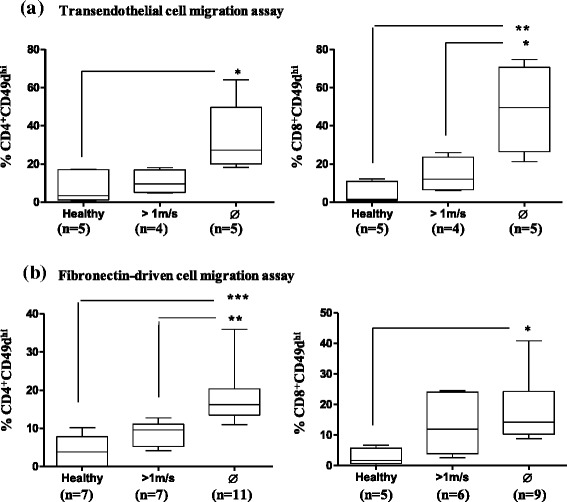
Enhanced migratory response of T lymphocytes from DMD patients correlates with disease severity. **a** Migration of T lymphocytes through endothelial cells. **b** Fibronectin-driven migration. Relative numbers of CD49d^hi^CD4^+^ and CD49d^hi^CD8^+^ migrating cells in healthy controls and in the different groups of DMD patients, subdivided according to their ability to walk. The number of DMD patients analyzed in each group appears in *parentheses*. *p < 0.05, **p < 0.01, ***p < 0.001

We next investigated the transendothelial migration of CD49d-positive T cells isolated from the different sub-groups of DMD patients with different disease severity. We observed that transendothelial migration of CD49d^hi^CD4^+^ and CD49d^hi^CD8^+^ T cells was higher when isolated from DMD patients than from their healthy counterparts (*p* < 0.05 and *p* < 0.01, respectively). More importantly, we observed that CD49d^hi^CD8^+^ T cells from wheelchair-bound DMD patients migrated faster through an endothelial layer than those isolated from less severe DMD patients, still able to walk faster than 1 m/s (*p* < 0.05) (Fig. 3a).

Since CD49d is a fibronectin receptor, we tested if its increase was functional by investigating whether T lymphocytes from DMD patients would migrate faster through a fibronectin lattice. Higher fibronectin-driven CD4^+^ T cell migratory responses were positively correlated with disease progression (*p* < 0.01) (Fig. 3b), being statistically significant for CD49d^hi^CD4^+^ and CD49d^hi^CD8^+^ T cells (*p* < 0.001 and *p* < 0.05, respectively) (Fig. 3b) when compared to healthy controls.

Considering that integrins are involved in leukocyte proliferation and survival [12], and that VLA-4 interaction with fibronectin induces lymphocyte proliferation, we evaluated cell proliferation and cell death after the 16-h migration period in the different groups of patients. No differences were observed (data not shown), indicating that the increased migratory responses are not related to increased proliferation or cell survival.

### Anti-CD49d mAb blocks transendothelial, fibronectin-driven migration and myotube adhesion of T cells from DMD patients

**Fig. 4:**
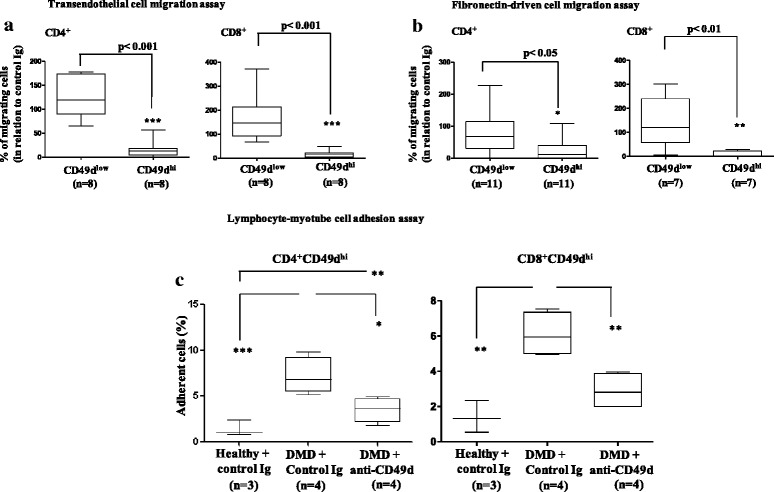
Ex vivo anti-CD49d antibody treatment preferentially blocks migratory responses of DMD-derived CD49d^hi^ T cells. Blocking effects of the anti-CD49d mAb upon transendothelial (**a**) and fibronectin-driven migration (**b**) of CD49d^low^CD4^+^ or CD49d^hi^CD4^+^ and CD49d^low^CD8^+^ or CD49d^hi^CD8^+^ T cells from DMD patients. Control Ig-treated cells were taken as 100 % of migratory/adhesive responses. **c** Adhesion onto myotubes of CD49d^hi^CD4^+^ and CD49d^hi^CD8^+^ T cells from DMD patients and healthy controls following treatment with anti-CD49d mAb or control Ig. *p < 0.05, **p < 0.01, ***p < 0.001

Because the VLA-4/VCAM-1 interaction on the surface of endothelial cells is important for transmigration of T cells from the blood, and considering that the VLA-4/fibronectin interaction is important for cell migration within the tissues, we investigated whether we could abrogate the migration of T cells ex vivo by selectively blocking CD49d. We pretreated T cells with an anti-CD49d mAb and compared their migration with that of T cells treated with an unrelated isotype-matched Ig. Anti-CD49d pretreatment efficiently and preferentially blocked transendothelial (*p* < 0.001) and fibronectin-driven migration of CD49d^hi^CD4^+^ (*p* < 0.05) and CD49d^hi^CD8^+^ (*p* < 0.01) T cells isolated from DMD patients (Fig. 4a, b). Moreover, since DMD-derived CD49d^hi^CD4^+^ (*p* < 0.001) and CD49d^hi^CD8^+^ (*p* < 0.01) T cells exhibited a higher adhesion onto monolayers of human myotubes (Fig. 4c), we tested the effect of blocking CD49d (Additional file 7: Figure S4a). The anti-CD49 mAb treatment significantly abrogated CD49d^hi^CD4^+^ and CD49d^hi^CD8^+^ T cell adhesion (*p* < 0.05 and *p* < 0.01, respectively), and in the case of CD8^+^CD49d^hi^ T cell subsets, values were similar to those seen when control Ig was applied on lymphocytes from healthy individuals (Fig. 4c). Moreover, anti-CD49d pretreatment preferentially blocked the adhesion of CD49d^hi^CD8^+^ (p=0.02) T cells (Additional file 7: Figure S4b). These results provide a proof of concept to design a therapeutic intervention to minimize the accelerating effect of increased CD49d^hi^ T cells, and of exacerbated inflammation, on the progression of the DMD phenotype.

## Discussion

We demonstrate that circulating CD49d^hi^ T cells can be successfully used as a biomarker for disease progression in DMD patients. Moreover, this seems to be specific for DMD since it is not found in IBM patients, who also suffer from an inflammatory muscle disease.

The percentages of circulating CD49d^hi^CD4^+^ and CD49d^hi^CD8^+^ T lymphocytes in DMD subjects correlated with disease prognosis in both ambulant and non-ambulant patients. Importantly, rapid disease progression in DMD patients can be predicted by detecting increased numbers of circulating CD49d^hi^CD4^+^ and CD49d^hi^CD8^+^ T lymphocytes at the beginning of the disease. This is also in agreement with our previous report, showing that decreased stride frequency and increased CD49d^hi^CD4^+^T lymphocytes are predictive markers of clinical outcome in the GRMD dog model of DMD [21]. Therefore, we propose that for the fast progressors, CD49d can be a biomarker for the prognosis and also for disease severity, since the frequencies of CD49d^hi^ T cells increase progressively once the patient begins to walk in 1 m/s or less. This fits with the hypothesis that CD49d can be involved in the pathogenesis of the muscular lesions.

Corticotherapy is widely used to improve DMD phenotype, and the chronic use of corticotherapy could modify the percentages of the CD49d^hi^ T cells. However, this is not the case since the patients who were no longer able to walk had stopped steroid treatment before the analyses, and the changes in CD49d expression persisted. In addition, it has been reported that glucocorticoids do not modulate in vitro or in vivo CD49d expression [22, 23]. Furthermore, all patients who were still able to walk were all under steroid treatment, thus excluding steroid treatment as a putative confounder. Yet, one can argue that good responders to glucocorticoids have a lower degree of inflammation and, as a consequence, lower levels of CD49d^hi^ T cells. When we followed the same patient at two different time points of disease (≤1 m/s: all under with corticotherapy, and unable to walk: with no longer corticosteroid treatment), we clearly found that the frequency of CD49d^hi^CD4+ T cells increased along with progression of severity only in the fast progressors. Considering that both groups (fast and slow progressors) were under the same conditions regarding corticotherapy, it suggests that the frequency of CD49d^hi^CD4^+^ T cells is not influenced by this drug. This individual follow-up with a small number of patients is supported by the same analysis in a transversal study with a larger cohort (Additional file 6: Figure S3a, b, e, f). CD49d can thus be used for clinical trials to stratify DMD patients into predictive slow and rapid progressors, whether these patients are under steroid treatment or not.

Using the T cell activation marker HLA-DR, we found a positive correlation with the relative number of CD49d^hi^HLA-DR^+^CD8^+^ T lymphocytes in the blood of patients who became wheelchair bound before 10 years of age. We also found more activated CD49d^+^HLA-DR^+^CD8^+^ T cells in the muscle of rapid progressors. Together, these findings support the concept that this subpopulation plays an important role in the pathogenesis of DMD and that the higher the relative number of CD49d^hi^ T cells in the blood, the more rapid is the disease progression.

More importantly, we show that both transendothelial and fibronectin-driven migration of CD49d^hi^ T cells from DMD patients were enhanced, as well as the binding of CD49d^hi^ T lymphocytes to myotubes. Considering that there was a correlation between the highest numbers of CD49d^hi^ T cells, the poor prognosis and disease severity, the highest migration, and the already known function of CD49d in the interaction with endothelium and fibronectin, we propose as an hypothesis that CD49d may facilitate T cell migration into muscle tissue. This suggests that CD49d acts by accelerating the migration of the T cells into the muscle, consequently enhancing inflammatory infiltrate in dystrophic muscle and directly targeting muscle fibers. Pretreatment of DMD T cells with an anti-CD49d mAb prevented transendothelial migration and decreased the ability of these cells to attach to myotubes. These findings strongly suggest that treatment with an anti-CD49d mAb may decrease in vivo the transendothelial migration of the T cells, impair their interaction with fibronectin within skeletal muscle, and abrogate cell-cell adhesive interactions with muscle fibers to slow down eventual necrosis.

It is important to mention that the pretreatment with anti-CD49d decreased the transendothelial and fibronectin-driven migration of CD49d^hi^ T cells but increased migration of CD49d^low^ T lymphocytes. In principle, cells with low expression of CD49d represent T cells that are not activated. In this regard, the influx into the muscle would not be a problem since non-activated cells would recirculate. In a second vein, CD49d^low^CD4^+^ T lymphocytes may correspond to regulatory T cells. If this is the case, they will rather have a protective anti-inflammatory role upon the entrance of activated CD49d^hi^HLA-DR^+^CD4^+^ T lymphocytes. Of note, we did not observe the same kind of event when lymphocytes were led to adhere onto myotubes. In those sets of experiments, no enhancement in the adhesion was observed regarding the CD49d^low^ T cells. Actually, a blockade of both low and high subpopulations seemed to occur after the pretreatment in vitro, being however more important in CD49d^hi^ T cells when compared to the corresponding low subpopulation. No difference was observed between low and high CD4^+^ T cells. Yet, considering the short number of patients studied, more experiments should be done to clarify this point.

The anti-CD49d mAb *Natalizumab* has already been used to treat multiple sclerosis and inflammatory bowel disease [24] and has proved to be beneficial for several thousands of patients [25–27], even though progressive multifocal leukoencephalopathy was reported in 0.08–0.03 % of patients treated for at least 24 months. *Natalizumab* has been well tolerated by children suffering from multiple sclerosis, resulting in clinical benefits [28]. Since DMD patients have high numbers of circulating CD49d^hi^ T cells as well as of CD49d^+^ T cells within the muscle, the therapeutic targeting of CD49d could be beneficial, and clinical trials should be envisaged to confirm this effect.

## Conclusions

We propose that CD49d can be used as a novel prognostic biomarker to stratify DMD patients into homogeneous cohorts in future clinical trials, improving potentially the significance of these costly studies. Inhibition of CD49d-mediated interactions could be envisioned as a novel therapeutic strategy for improving disease progression in DMD patients, by decreasing the immune response, in conjunction with other gene therapy approaches such as exon skipping or gene replacement, which in some cases triggers dystrophin-specific immune response [29–31].

## Additional files

Additional file 1: Table S1. General characteristics of the DMD patients enrolled in the study of blood samples. (DOC 35 kb) Additional file 2: Table S2. Age-related features of DMD patients enrolled in the evaluation of muscle biopsies. (DOC 37 kb) Additional file 3: Table S3. Antibodies applied in cytofluorometry and immunohistochemistry. (DOC 57 kb) Additional file 4: Figure S1. Gating procedures for cytofluorometric labeling of CD49d in freshly isolated leukocytes from the blood of normal subjects and Duchenne muscular dystrophy patients. (DOC 108 kb) Additional file 5: Figure S2. Lack of differences between DMD patients and healthy control in relative numbers of CD14/CD49d^hi^ and CD19/CD49d^hi^ cells. (DOC 69 kb) Additional file 6: Figure S3. Higher numbers of CD49d^hi^ T cells in fast, but not in slow, progressors DMD patients correlate with disease severity. (DOC 59 kb) Additional file 7: Figure S4. Ex vivo anti-CD49d antibody treatment blocks lymphocyte-myotube adhesion in DMD patients. (DOC 520 kb)

## Abbreviations

DMD: Duchenne muscular dystrophy
ECM: extracellular matrix
Ig: immunoglobulin
mAb: monoclonal antibody
VLA: very late antigen
