# Supplementary material for: CD49d is a disease progression biomarker and a potential target for immunotherapy in Duchenne muscular dystrophy
Source: Skelet Muscle. 2015 Dec 10;5:45. doi: 10.1186/s13395-015-0066-2 (PMC4674917; doi:10.1186/s13395-015-0066-2)
Supplement: Additional file 1: Table S1. — General characteristics of the DMD patients enrolled in the study of blood samples. (DOC 35 kb) [file 13395_2015_66_MOESM1_ESM.doc]

**Additional file Table 1. General characteristics of the DMD patients enrolled in the study of blood samples**

|  | | **DMD patients^a,b^** | | | **Healthy Controls** |
| --- | --- | --- | --- | --- | --- |
|  | | ***>1m/s*** | ***≤1m/s*** | ***Unable to walk*** |  |
| **Numbers (%)** | | 19 (25.33) | 25 (33.33) | 31 (41.33) | 14 |
|  | *Minimum* | 5 | 6 | 7 | 4 |
| **Age (yr)** | *Median (IQR)* | 6 (1) | 9 (3.5) | 13.5 (6.5) | 10 (8.7) |
|  | *Mean + SD* | 6.4+1.12 | 8.5+1.80 | 12·5+3.44 | 10.9+5.48 |
|  | *Maximum* | 10 | 11 | 17 | 20 |

**^a^**All patients were treated with prednisone (1mg/kg/day during the first 10 days of each month) from the time at which the diagnosis was confirmed until they were wheel chair bound, when treatment was stopped. **^b^**Patients and controls with any co-morbidity that could interfere with the immunologic status were not enrolled in the study. The co-morbidities were defined by analyzing the clinical history of the patients, clinical examination and laboratory exams.
