# Supplementary material for: CD49d is a disease progression biomarker and a potential target for immunotherapy in Duchenne muscular dystrophy
Source: Skelet Muscle. 2015 Dec 10;5:45. doi: 10.1186/s13395-015-0066-2 (PMC4674917; doi:10.1186/s13395-015-0066-2)
Supplement: Additional file 2: Table S2. — Age-related features of DMD patients enrolled in the evaluation of muscle biopsies. (DOC 37 kb) [file 13395_2015_66_MOESM2_ESM.doc]

**Additional file table 2. Age-related features of DMD patients enrolled in the evaluation of muscle biopsies***

| **Patient** | **Age at biopsy (years)** | **Age at loss of ambulation (years)** | **Time passed between biopsy and loss of ambulation (years)** |
| --- | --- | --- | --- |
| 1 | 3 | < 10 | 6 |
| 2 | 4 | ≥ 10 | 7·5 |
| 3 | 7 | < 10 | 1 |
| 4 | 7 | ≥ 10 | 4 |
| 5 | 3 | < 10 | 4·5 |
| 6 | 7 | < 10 | 2 |
| 7 | 5 | ≥ 10 | 5·5 |
| 8 | 5 | ≥ 10 | 5·5 |
| 9 | 5.5 | ≥ 10 | 7 |

*The biopsies were carried out between 3-7 years of age, and there was no significant difference in the age between the groups (p=0·90). The mean time from biopsy to the loss of ambulation was 3.3 years (± 1·14) and 5·9 years (± 0·62), in the groups that lost the ability to walk before or after 10 years of age, respectively. There was no significant difference between the groups (p = 0·17). They were not receiving corticosteroid treatment at the time of the study, and the muscle biopsies were obtained as part of the routine diagnostic procedure (at the time of diagnosis of the disease), with informed consent. The choice of the muscle for the biopsy, usually the quadriceps, was based on the physical examination, and this muscle should be able to generate a force of at least 3/5 according to the MRC (Medical Research Council, UK) scale. This scale corresponds to scores varying from zero to five: ***0.*** no movement; ***1****.* flicker is perceptible in the muscle; ***2****.* movement only if gravity eliminated; ***3****.* the patient can move limb against gravity; **4**. the patient can move against gravity & some resistance exerted by examiner; ***5****.* normal power.
