# Supplementary material for: CD49d is a disease progression biomarker and a potential target for immunotherapy in Duchenne muscular dystrophy
Source: Skelet Muscle. 2015 Dec 10;5:45. doi: 10.1186/s13395-015-0066-2 (PMC4674917; doi:10.1186/s13395-015-0066-2)
Supplement: Additional file 3: Table S3. — Antibodies applied in cytofluorometry and immunohistochemistry. (DOC 57 kb) [file 13395_2015_66_MOESM3_ESM.doc]

**Additional file table 3. Antibodies applied in cytofluorometry and immunohistochemistry**

| **Primary antibodies** | | | | | |
| --- | --- | --- | --- | --- | --- |
| ***Molecular specificity*** | ***Species specificity*** | ***Species origin and immunoglobulin isotype*** | ***Fluorochrome*** | ***Clone*** | ***Manufacturer*** |
| CD3 | Human | Mouse mAb* IgG1 | PercP | SK7 | BD Pharmingen, Franklin Lakes, NJ, USA |
| CD4 | Human | Mouse mAb IgG1 | Alexa Fluor 488 | RPA-T4 | BD Pharmingen, Franklin Lakes, NJ, USA |
| CD8 | Human | Mouse mAb IgG1 | Alexa Fluor 647 | RPA-T8 | BD Pharmingen, Franklin Lakes, NJ, USA |
| CD19 | Human | Mouse mAb IgG1 | PercP | 4G7 | BD Pharmingen, Franklin Lakes, NJ, USA |
| CD14 | Human | Mouse mAb IgG2a | FITC* | M5E2 | BD Pharmingen, Franklin Lakes, NJ, USA |
| CD11a | Human | Mouse IgG1 | PE* | HI111 | BD Pharmingen, Franklin Lakes, NJ, USA |
| CD49a | Human | Mouse mAb IgG1 | PE | SR84 | BD Pharmingen, Franklin Lakes, NJ, USA |
| CD49d | Human | Mouse mAb IgG1 | PE | 9F10 | BD Pharmingen, Franklin Lakes, NJ, USA |
| CD49e | Human | Mouse mAb IgG1 | PE | IIA1 | BD Pharmingen, Franklin Lakes, NJ, USA |
| CD49f | Human | Rat mAb IgG2a | PE | GoH3 | BD Pharmingen, Franklin Lakes, NJ, USA |
| CD3 | Human | Rabit polyclonal antibody | ------------ | ------------ | Dako, Trappes, France |
| CD4 | Human | Mouse mAb IgG1 | ------------ | MT310 | Dako, Trappes, France |
| CD8 | Human | Mouse mAb IgG1 | ------------ | C8/144B | Dako, Trappes, France |
| HLA-DR | Human | Mouse mAb IgG2b | ------------ | EDU-1 | Invitrogen, Cergy Pontoise, France |
| CD49d | Human | Mouse mAb IgG3 | ------------ | P4C2 | Abcam, Paris, France |
| Fibronectin | Human | Rabbit polyclonal antibody | ------------ | ------------ | Dako, Trappes, France |
| **Secondary antibodies** | | | | | |
| Mouse IgG1 | Mouse | Goat polyclonal antibody | Alexa Fluor 594 | ------------- | Jackson Immunoresearch, Suffolk, UK |
| Mouse IgG2b | Mouse | Goat polyclonal antibody | Alexa Fluor 488 | ------------- | Molecular Probes, Cergy Pontoise, France |
| Mouse IgG3 | Mouse | Goat polyclonal antibody | Biotinylated | ------------- | Jackson Immunoresearch, Suffolk, UK |
| Rabbit Ig | Rabbit | Goat polyclonal antibody | Cy3; Alexa Fluor 488 | ------------- | Jackson Immunoresearch, Suffolk, UK |
| **Antibody for blocking assays** | | | | | |
| CD49d | Human | Mouse mAb IgG1 | ------------ | 2B4 | R&D System, Lille, France |

*MAb: monoclonal antibody; FITC: fluoresceinisthyocyanate; PE: phycoerythin.
