# Supplementary material for: CD49d is a disease progression biomarker and a potential target for immunotherapy in Duchenne muscular dystrophy
Source: Skelet Muscle. 2015 Dec 10;5:45. doi: 10.1186/s13395-015-0066-2 (PMC4674917; doi:10.1186/s13395-015-0066-2)
Supplement: Additional file 4: Figure S1. — Gating procedures for cytofluorometric labeling of CD49d in freshly isolated leukocytes from the blood of normal subjects and Duchenne muscular dystrophy patients. (DOC 108 kb) [file 13395_2015_66_MOESM4_ESM.doc]

**Additional file figure 1. Gating procedures for cytofluorometric labeling of CD49d in freshly-isolated leukocytes from the blood of normal subjects and Duchenne muscular dystrophy patients.** Mononuclear cells obtained after ficoll-hypaque isolation were first analyzed in terms of forward scatter (*FSC*) versus side scatter (*SSC*). Gates were done in the typical lymphocyte FSC x SSC pattern and analyzed for the labeling for CD3 *versus* CD4 or CD8. Those CD3^+^CD4^+^ as well as CD3^+^CD8^+^ cells were further gated for definition of CD49d labeling. CD49d^+^ lymphocytes were defined after comparing with the fluorescent signal generated by the corresponding isotype/fluorochrome-matched unrelated immunoglobulin, applied at the same concentration. Among the CD49d-positive cells within the CD4^+^ and CD8^+^ subsets, we could identify low and high CD49d expressors, displayed in the figure separated by dashed lines.
