# Supplementary material for: CD49d is a disease progression biomarker and a potential target for immunotherapy in Duchenne muscular dystrophy
Source: Skelet Muscle. 2015 Dec 10;5:45. doi: 10.1186/s13395-015-0066-2 (PMC4674917; doi:10.1186/s13395-015-0066-2)
Supplement: Additional file 5: Figure S2. — Lack of differences between DMD patients and healthy control in relative numbers of CD14/CD49dhi and CD19/CD49dhi cells. (DOC 69 kb) [file 13395_2015_66_MOESM5_ESM.doc]

**Additional file figure 2. Lack of differences between DMD patients** **and healthy control in relative numbers of CD14/CD49d^hi^ and CD19/CD49d^hi^ cells.** Monocytes were detected by an anti-CD14 monoclonal antibody whereas B lymphocytes were defined by the expression of CD19. Upper panels depict histograms of CD49d membrane expression in CD19^+^ and CD14^+^ cells in healthy subjects and DMD patients. Bars represent mean + standard error of the relative number of labeled cells, and *p* values are shown within each graphic.
