# Supplementary material for: CD49d is a disease progression biomarker and a potential target for immunotherapy in Duchenne muscular dystrophy
Source: Skelet Muscle. 2015 Dec 10;5:45. doi: 10.1186/s13395-015-0066-2 (PMC4674917; doi:10.1186/s13395-015-0066-2)
Supplement: Additional file 6: Figure S3. — Higher numbers of CD49dhi T cells in fast, but not in slow, progressors DMD patients correlate with disease severity. (DOC 59 kb) [file 13395_2015_66_MOESM6_ESM.doc]

**Additional file figure 3. Higher numbers of CD49d^hi^ T cells in fast, but not in slow, progressors DMD patients correlate with disease severity.** Relative number of CD4^+^ and CD49d^hi^CD8^+^ T cells in different groups of DMD patients organized according to their disease progression (≤1m/s and unable to walk). CD49d^hi^CD4^+^ T cells were analysed in a transversal study of slow progressors **(a)** and fast progressors **(b)** patients subdivided according to disease severity (able to walk in ≤1m/s and wheel chair bound patients). The same was performed for CD49d^hi^CD8^+^ T cells **(e, f).** In a longitudinal study the same patient was followed up during the disease progression (**c, d**). In (**c**) and (**d**) the relative numbers of CD49d^hi^CD4^+^ T cells were analysed in the slow and fast progressors respectively.
