# Supplementary material for: CD49d is a disease progression biomarker and a potential target for immunotherapy in Duchenne muscular dystrophy
Source: Skelet Muscle. 2015 Dec 10;5:45. doi: 10.1186/s13395-015-0066-2 (PMC4674917; doi:10.1186/s13395-015-0066-2)
Supplement: Additional file 7: Figure S4. — Ex vivo anti-CD49d antibody treatment blocks lymphocyte-myotube adhesion in DMD patients. (DOC 520 kb) [file 13395_2015_66_MOESM7_ESM.doc]

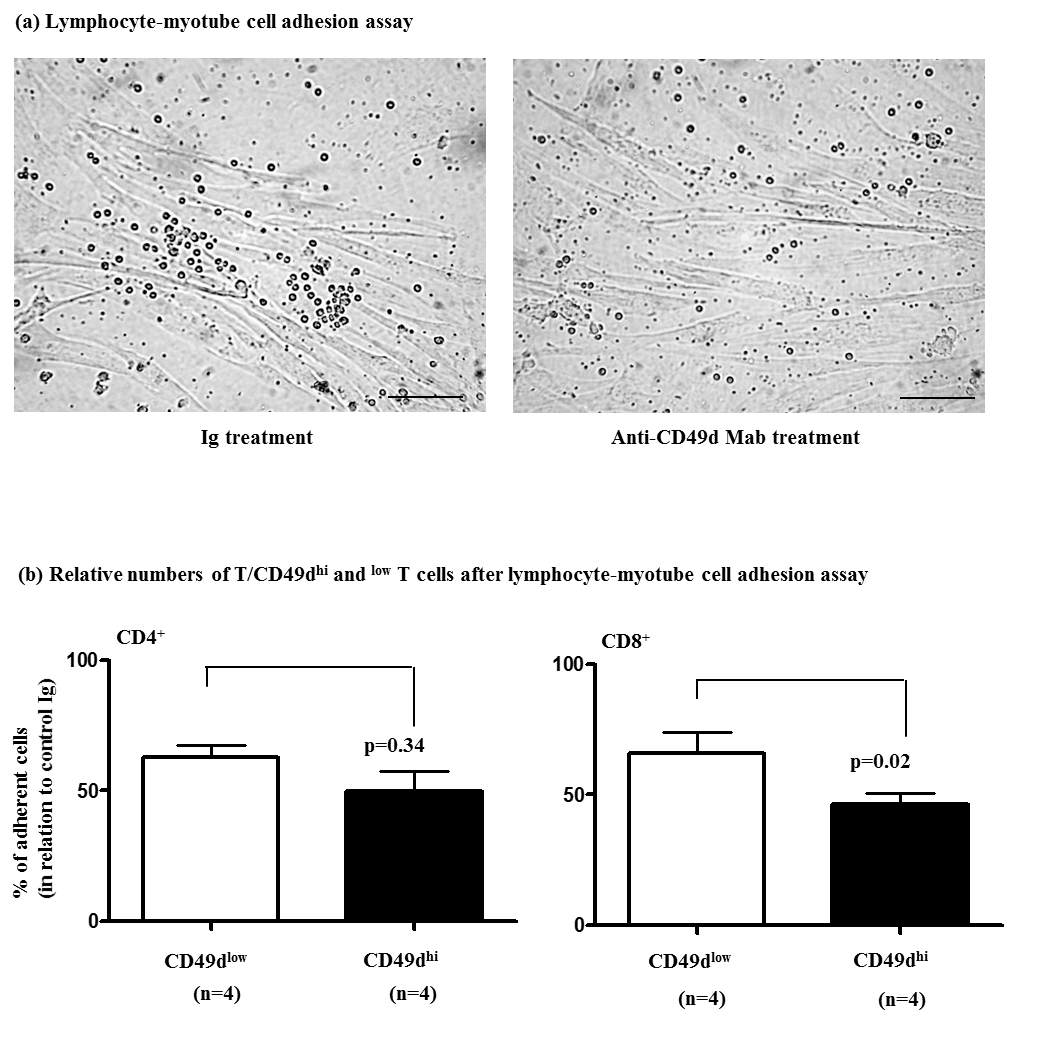


**Additional file figure 4. *Ex-vivo* anti-CD49d antibody treatment blocks lymphocyte-myotube adhesion in DMD patients.** A representative picture of the adhesion of the lymphocytes on myotubes after the treatment with irrelevant isotype control and with monoclonal antibody anti-CD49d can be seen in (**a**). In (**b**) control Ig-treated cells were taken as 100% of adhesive response, and the adhesion to myotubes of T/CD49d^hi^ and T/CD49dl^ow^ cells from DMD patients following treatment with anti-CD49d mAb relative numbers of the adherent lymphocytes comparing. Bars= 50µm.
